# Defibrotide enhances fibrinolysis in human endotoxemia – a randomized, double blind, crossover trial in healthy volunteers

**DOI:** 10.1038/s41598-019-47630-6

**Published:** 2019-07-31

**Authors:** Christian Schoergenhofer, Nina Buchtele, Georg Gelbenegger, Ulla Derhaschnig, Christa Firbas, Katarina D. Kovacevic, Michael Schwameis, Philipp Wohlfarth, Werner Rabitsch, Bernd Jilma

**Affiliations:** 1 Department of Clinical Pharmacology, Medical University of Vienna, Wien, Austria; 2 Department of Emergency Medicine, Medical University of Vienna, Wien, Austria; 3 Department of Blood and Bone Marrow Transplantation, Medical University of Vienna, Wien, Austria

**Keywords:** Experimental models of disease, Translational research

## Abstract

Defibrotide is approved for the treatment of sinusoidal obstruction syndrome after allogeneic stem cell transplantation. The exact mode of action of defibrotide is unclear and human *in vivo* data are scarce. In this randomized, double blind, crossover trial we included 20 healthy volunteers. Four were randomized to receive placebo, while 16 received a 2 ng/kg bodyweight bolus of lipopolysaccharide (LPS). Infusion of 6.25 mg/kg defibrotide or placebo was started one hour before the injection of the LPS bolus. Plasma levels of prothrombin fragments F1 + 2, thrombin-antithrombin complexes, von Willebrand factor, E-selectin, tissue-type plasminogen activator (t-PA), plasminogen activator inhibitor-1 (PAI-1), plasmin-antiplasmin complexes (PAP), tumor necrosis factor-α, interleukin 6, and C-reactive protein were measured. Thromboelastometry was performed. Infusion of defibrotide did not reduce the LPS-induced activation of coagulation, the endothelium or the release of pro-inflammatory cytokines. However, defibrotide increased t-PA antigen levels by 31% (Quartiles: 2–49%, p = 0.026) and PAP concentrations by 13% (−4–41%, p = 0.039), while PAI-1 levels remained unaffected. Moreover, defibrotide reduced C-reactive protein levels by 13% (0–17%, p = 0.002). A transient increase in the clotting time in thromboelastometry and a decrease in F1 + 2 prothrombin fragments suggests modest anticoagulant properties. In conclusion, defibrotide infusion enhanced fibrinolysis and reduced C-reactive protein levels during experimental endotoxemia.

## Introduction

Defibrotide is a highly complex polydisperse mixture of single-stranded phosphodiester oligodeoxyribonucleotides derived from the controlled depolymerization of porcine intestinal mucosal DNA^1^. Defibrotide is approved for use in sinusoidal obstruction syndrome, which mainly occurs after high dose chemotherapy in the setting of allogeneic hematopoietic stem cell transplantation^2^. In the pivotal trial defibrotide improved 100-day survival in patients with sinusoidal obstruction syndrome compared to historical controls from 25% to 38%^3^. In a randomized, placebo-controlled phase 3 trial defibrotide was effective in the prophylaxis of sinusoidal obstruction syndrome and reduced its incidence from 20% to 12%^4^. Interestingly, in this trial defibrotide also reduced the development of acute graft versus host disease (GVHD) from 52 to 34%^4^.

However, the mode of action of defibrotide remains largely unknown and it was reported to induce multiple anti-inflammatory, pro-fibrinolytic and anticoagulant effects^5^. Defibrotide dose dependently increased t-PA activity and antigen, but reduced PAI-1 and tissue factor expression *in vitro*^6^. Furthermore, defibrotide reduced tumor necrosis factor (TNF)-α expression in lipopolysaccharide (LPS)-stimulated dendritic cells and it attenuated the prothrombinase activity, platelet and complement activation in *in vitro* malaria models^7^. In healthy volunteers, combined infusion of defibrotide and heparin significantly enhanced the effect of heparin alone and prolonged the activated partial thromboplastin time and increased antithrombin III levels^8^. Moreover, defibrotide inhibited factor Xa, increased tissue factor pathway inhibitor and decreased tissue factor in healthy volunteers^9^. However, human *in vivo* data are scarce and the exact mode of action remains to be elucidated.

The human endotoxemia model is an established model of a tissue-factor driven, self-limiting, acute inflammatory reaction in healthy human volunteers^10,11^. After a bolus infusion of LPS pro-inflammatory cytokines increase, which is accompanied by an activation of the coagulation system, fibrinolysis and of the endothelium^10,12^.

The aim of the current trial was to better define the effects of defibrotide *in vivo* using approved doses in an established model of acute inflammatory response in healthy volunteers^10,11^. The ascribed profibrinolytic and antithrombotic properties of defibrotide are mostly based on (i) *in vitro* experiments^6,7^, in which exceedingly high concentrations of defibrotide have been used compared to those concentrations found after infusion of approved doses^13,14^, (ii) non-randomized studies in patients undergoing hematopoietic stem cell or bone marrow transplantation^15,16^, or (iii) studies in healthy volunteers using bolus infusions or much higher doses than currently approved^8,9,17^. However, currently no such data exist for the approved doses and therefore it is unclear whether these effects are translatable in full extent. Moreover, in contrast to *in vitro* studies, human *in vivo* trials allow investigating the effects of defibrotide in the complex interplay of inflammation, coagulation, fibrinolysis and endothelial activation.

We hypothesized that defibrotide diminishes the pathophysiological changes caused by experimental endotoxemia.

## Results

Twenty healthy volunteers, two females and 18 males, were included in this trial with a median age of 28.5 years (Quartiles: 24–34 years), a median height of 177 cm (174–183 cm), and a median weight of 72 kg (66–84 kg). One person randomized to the placebo group did not participate in the second period due to unexpected unavailability. In one person in the placebo period infusion of defibrotide was stopped due to the development of an urticarial rash.

### *Ex vivo* study

**Figure 1:**
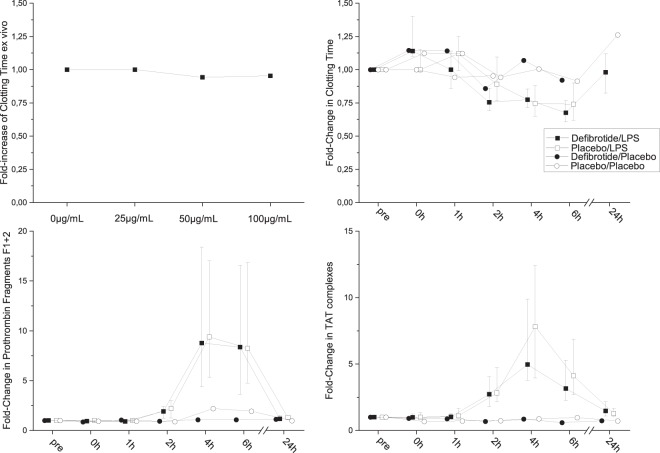
Changes in Coagulation Specific Parameters. Left upper panel: Results of *ex-vivo* spiking study: clotting time was measured in whole blood spiked with various concentrations of defibrotide (0 µg/mL, 25 µg/mL, 50 µg/mL and 100 µg/mL; n = 8); right upper panel: clotting time measured by thromboelastometry during experimental endotoxemia; left lower panel: prothrombin complex F1 + 2 concentrations during experimental endotoxemia; right lower panel: fold-change in thrombin antithrombin complex levels during experimental endotoxemia and in the placebo period (n = 16 for LPS, n = 4 for placebo); Presented are medians ± interquartile range.

The addition of defibrotide to whole blood did not affect the results of thromboelastometry. No dose dependent effects were measured with regards to clotting time or maximum lysis (Fig. 1).

### Coagulation

As expected, endotoxemia increased *in vivo* thrombin generation (prothrombin fragments F1 + 2 and TAT complexes), and thromboelastometry confirmed shortened clotting times (Fig. 1).

There was no difference in prothrombin fragments F1 + 2 between both study periods. The ratio of the AUCs for both periods showed a numerical 13% reduction in the defibrotide period (median 0.87, quartiles: 0.75–1.1, p = 0.38, n = 16). Also TAT levels did not differ between both trial periods. The ratio of the AUCs was 0.83 (0.56–0.9, p = 0.15, n = 16) indicating a numerical reduction (Fig. 1). Defibrotide infusion did not prevent the shortening in the clotting time compared to placebo during endotoxemia. This parameter reflects the activation of coagulation. Interestingly, in an exploratory analysis the clotting time increased transiently 1 h after start of the defibrotide infusion by a median of 12% (quartiles: 0–30%, p = 0.003, Fig. 1, n = 16). This is also reflected by a small 8% decrease in F1 + 2 prothrombin fragments from baseline (p = 0.036). Of note, in the placebo period a trend to an increased clotting time could also be observed 1 h vs. 2 h after start of the infusion. Defibrotide did not prevent the LPS-induced decrease in platelet counts, which decreased in both periods by approximately 20% (Supplementary Information, Figure S1). 

Maximum concentrations of F1 + 2 correlated with maximum TAT concentrations (r = 0.59, p < 0.001, n = 16).

### Fibrinolysis

**Figure 2:**
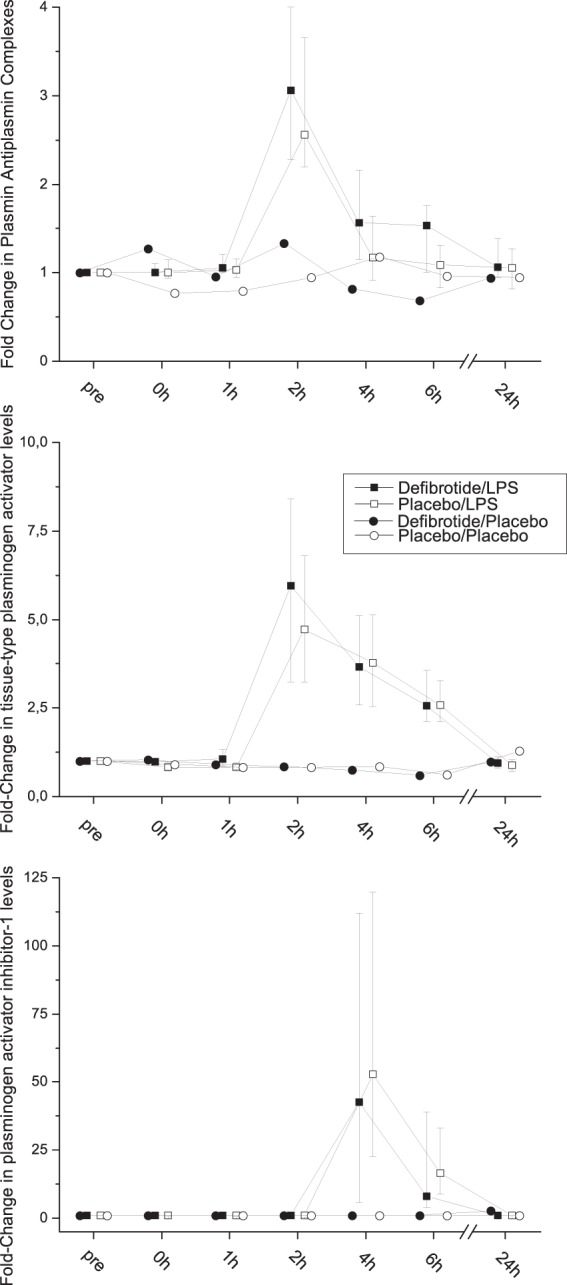
Fibrinolysis Specific Parameters. Upper panel: fold-change in plasmin-antiplasmin complex levels (n = 16 for LPS, n = 4 for placebo); middle panel: fold-change in tissue-type plasminogen activator levels (n = 16 for LPS, n = 4 for placebo); lower panel: fold-change in plasminogen activator inhibitor-1 levels during experimental endotoxemia and after placebo (n = 16 for LPS, n = 4 for placebo); Presented are medians ± interquartile range.

Infusion of LPS activated the fibrinolytic system in both trial periods (Fig. 2).

PAP complexes were higher in the defibrotide period (p = 0.039). The ratio of the AUC was 1.13 in the defibrotide period (quartiles: 0.96–1.41, Fig. 2, n = 16). This was accompanied by a more pronounced increase of t-PA antigen in the defibrotide period (p = 0.026, n = 16). The median ratio of the AUC of both periods was 1.31 (1.02–1.49, Fig. 2). There was no difference in PAI levels between both study periods with a median ratio of the AUC of 1.03% (0.32–1.58, p = 0.92; Fig. 2, n = 16). Maximum lysis in thrombelastometry did not differ between both study periods.

The relationship between fibrinolysis parameters and inflammation was examined by correlation analysis. Maximum values of ML correlated well with maximum PAP concentrations (r = 0.65, p < 0.001). Likewise ML correlated with maximum levels of tPA concentrations (r = 0.41, p = 0.02) and with maximum TNF-α concentrations (r = 0.52, p = 0.02). Maximum tPA concentrations correlated well with maximum TNF-α concentrations (r = 0.68, p < 0.001). Maximum TNF-α concentrations also correlated with maximum PAP concentrations (r = 0.45, p = 0.009) and with maximum PAI concentrations (r = 0.45, p = 0.011).

### Inflammation

**Figure 3:**
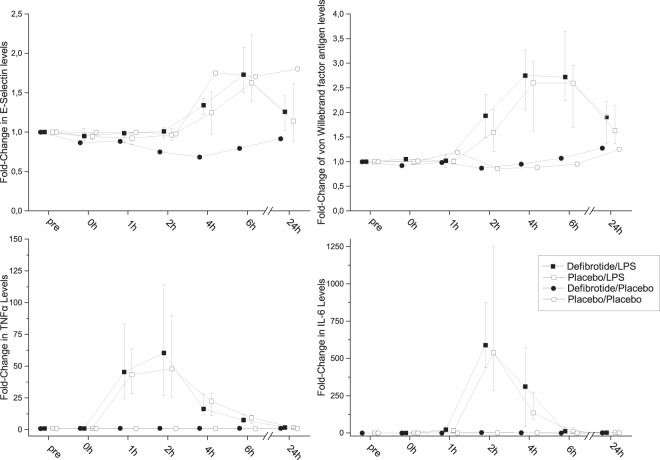
Endothelium specific parameters and pro-inflammatory cytokines. Left upper panel: fold-change in E-selectin concentrations during experimental endotoxemia (n = 16 for LPS, n = 4 for placebo); right upper panel: fold-change in von Willebrand factor levels during experimental endotoxemia (n = 16 for LPS, n = 4 for placebo); left lower panel: fold-change TNF-α concentrations during experimental endotoxemia and after infusion of placebo (n = 16 for LPS, n = 4 for placebo); right lower panel: fold-change in interleukin-6 levels during experimental endotoxemia (n = 16 for LPS, n = 4 for placebo); Presented are medians ± interquartile range.

There was no difference in pro-inflammatory cytokines IL-6 (median ratio 163% (64–231%), p = 0.33, n = 16) and TNF-α (median ratio 107% (72–138%), p = 0.5; Fig. 3, n = 16). Interestingly, C-reactive protein levels were lower in the defibrotide period (median ratio 0.87, quartiles 0.83–1, p = 0.021, n = 16). Defibrotide did not prevent the LPS-induced increase in total leukocyte counts, which increased approximately two-fold in both study periods (Supplementary information, Figure S1).

Maximum concentrations of IL-6 correlated well with C-reactive protein levels after 24 h (r = 0.57, p = 0.001) and with maximum TNF-α concentrations (r = 0.63, p < 0.001). Maximum TNF-α concentrations correlated also well with C-reactive protein levels after 24 h (r = 0.61, p < 0.001).

### Endothelium

There were no differences in biomarkers of endothelial activation. The ratio of the AUCs was in median 100% (80–116%, p = 0.88, n = 16) for vWF and 100% (77–114%, p = 0.8, n = 16) for E-sel (Fig. 3).

Maximum levels of E-sel correlated with maximum levels of vWF (r = 0.41, p = 0.019). Maximum TNF concentrations correlated with maximum levels of vWF (r = 0.58, p < 0.001) and of E-sel (r = 0.5 p = 0.004).

### Adverse events

Thirteen subjects (81%) in the defibrotide/LPS period reported a total of 29 adverse events, while in the placebo/LPS period 15 subjects (94%) reported 33 adverse events. The most frequent adverse events included headache (13 in defibrotide, 11 in placebo period), flu-like symptoms (6 in defibrotide, 6 in placebo period), arthralgia (2 in defibrotide, 7 in placebo period), chills or feeling cold (3 in defibrotide, 6 in placebo period), nausea (2 defibrotide, 1 placebo) with single episodes of impaired concentration (placebo), fatigue (placebo), precollapse (defibrotide), dizziness (defibrotide), vertigo (defibrotide). In the LPS periods subjects regularly received paracetamol for symptom relieve (17 × 500 mg defibrotide, 18 × 500 mg placebo), one subject received 10 mg of metoclopramide for nausea (defibrotide and placebo period), one subject received one tablet of metamizol 500 mg (defibrotide), and one subject received a combination of paracetamol 450 mg and orphenadrincitrate 35 mg for arthralgia. All adverse events were graded mild or moderate. There were no obvious differences in frequency or severity of adverse events between both trial periods.

In the placebo/placebo period one subject reported to have headache and received 500 mg paracetamol as treatment. One subject developed an urticarial rash during the defibrotide infusion in the placebo period, the infusion was stopped and she was treated with 30 mg diphenhydramine and 50 mg aprednisolone after which the rash resolved.

## Discussion

This was the first trial that investigated the effects of defibrotide on acute inflammatory responses in healthy human volunteers. The approved dosing regimen (infusion of 6.25 mg/kg bodyweight over 2 h) was used in our trial, which allows translation of the observed effects to the patient situation.

In the first part of the trial we investigated the effects of defibrotide on thromboelastometry *ex vivo*, while the second part studied the effects of defibrotide on experimental endotoxemia in healthy human volunteers *in vivo*. Our major findings were that (i) *ex vivo* defibrotide had no effects on thromboelastometr*y*; (ii) *in vivo*, defibrotide enhanced fibrinolysis, as indicated by increased PAP complexes and increased levels of tPA, (iii) defibrotide reduced C-reactive protein levels although pro-inflammatory cytokines remained unaffected, (iv) defibrotide did not impact on the activation of coagulation assessed by F1 + 2 prothrombin complexes, TAT complexes, and thromboelastometry, and (v) defibrotide did not reduce endothelial activation.

In our trial defibrotide infusion increased t-PA antigen levels by 30% and PAP complexes by 13% during endotoxemia, while PAI-1 levels remained unchanged. The induction of fibrinolysis is commonly attributed to treatment with defibrotide, although this is based only on few *in vivo* data. Coccheri *et al*. demonstrated the profibrinolytic effects of defibrotide in 1988 showing that defibrotide infusion in healthy human volunteers decreased PAI-1 levels by 80%, while t-PA increased almost five-fold. However, similar changes occurred in the placebo period raising concerns about the real effect of defibrotide in this trial. Noteworthy, in that earlier trial a defibrotide dose of approximately 15 mg/kg bodyweight was used, which was >2-fold higher compared to the dose used in our trial^18^. However, dose dependent effects of defibrotide on plasmin activity, the release of t-PA and reductions in PAI-1 were also shown *in vitro*^19–21^. In a model of endothelial cells stimulated with LPS defibrotide also induced profibrinolytic changes. However, PAI-1 and t-PA antigen showed marked responses only at defibrotide concentrations exceeding 200 µg/mL^6^. According to several pharmacokinetic studies, maximum concentrations in humans do not exceed 100 µg/mL using the approved dose of 6.25 mg/kg bodyweight^13,14^. Thus, although in principal our findings confirm these data, the fibrinolytic response may be more pronounced at higher doses. Clinical data report that PAI-1 antigen levels decrease over time in patients with sinusoidal obstruction syndrome who are successfully treated with defibrotide, whereas in patients who do not respond to treatment, PAI-1 levels remained stable or even increased^15,22^. No clinical data are available for using higher defibrotide doses in patients who do not respond to treatment according to PAI-1 concentrations, although *in vitro* data support such an approach.

Fibrinolysis is regulated by TNF-α in human endotoxemia: reduction or inhibition of TNF-α results in a shutdown of fibrinolysis^23,24^. In our trial TNF-α concentrations were only numerically higher in the defibrotide period compared to placebo. Thus, our findings contrast *in vitro* studies, in which defibrotide significantly reduced TNF-α concentrations upon LPS stimulation already at concentrations of 30 µg/mL^7,25^. Furthermore, the observed increases in PAP and t-PA are relatively small with uncertain clinical relevance. However, our trial was performed in healthy volunteers and not in severely ill patients with impaired coagulation parameters and thrombocytopenia^26,27^. Moreover, treatment of sinusoidal obstruction syndrome is usually performed over weeks and not only for a couple of hours^3,15,22^. Thus, although the observed effects on fibrinolysis are small, they may be of clinical relevance in patients with sinusoidal obstruction syndrome.

TNF-α is also critically involved in the regulation of E-sel expression and the release of vWF from endothelial cells. Inhibition or reduction of TNF reduced or blunted the endothelial response assessed by vWF or E-sel^23,28,29^. Defibrotide did not reduce TNF-α concentrations, therefore no differences in vWF or E-sel were found. Furthermore there was no difference in IL-6 levels between both trial periods. C-reactive protein is an inflammatory marker and is largely IL-6 dependent in this model^30^. Thus, this may reflect decreased IL-6 dependent effects and consequently reduced production of C-reactive protein by liver cells^31^. However, the observed difference in C-reactive protein levels is small and may also be due to chance.

Defibrotide had no impact on the LPS-induced activation of coagulation assessed by thromboelastometry *ex vivo* regardless of the dose. In additional *in vitro* experiments concentrations up to 1000 µg/mL had no direct effects on coagulation (data not shown). Thus, we assume that any effects of defibrotide on the coagulation are not direct, but are dependent on mediators released from the endothelium or other cell types not present in whole blood. Defibrotide had no influence on coagulation parameters during experimental endotoxemia *in vivo*. The transient prolongation of the clotting time of ~12% may reflect modest actions of defibrotide on coagulation also observed in the ~8% decrease in F1 + 2 concentrations. In patients who receive defibrotide for sinusoidal obstruction syndrome we have performed thromboelastometry to monitor its effects and we have observed more pronounced prolongations in the clotting time by approximately 15 to 35% between trough levels (before next infusion) and at maximum concentrations (at the end of the infusion, data not shown). Noteworthy, in our trial these effects were quite variable with a coefficient of variation of 128% between baseline and after an infusion time of 1 h. In five subjects we measured a >20% prolongation of the clotting time compared to baseline. The LPS induced activation of coagulation completely blunted this observed effect. Interestingly, bolus infusion of 400 mg defibrotide increased tissue factor pathway inhibitor approximately 2-fold, reduced tissue factor by 27% and showed pronounced anti-Xa activity in healthy volunteers, which is comparable to low molecular weight heparins^9^. However, the tissue-factor driven activation of coagulation during endotoxemia remained unaffected in our trial, which is in contrast to danaparoid^32^. Thus, we assume only modest effects of defibrotide on coagulation. The present data are insufficient to draw conclusions on dose-dependency of these effects. Thromboelastometry may be of value to monitor the effects of defibrotide, to identify patients at risk of bleeding in case of an overshooting response, but maybe also identify effective doses. However, clinical data are required to support this hypothesis.

### Limitations

The endotoxemia model involves healthy human volunteers and a bolus infusion of LPS to induce an acute inflammatory response, which naturally differs from the pathogenesis and the patient characteristics of sinusoidal obstruction syndrome. We have only used the authorized dose of defibrotide, although some of the effects may be dose-dependent and higher doses may be required in some subjects. The sample size was rather small and therefore small effects may not have been detected. We only infused a single dose of defibrotide, which contrasts the continuous treatment of patients for a period of three weeks. The chosen endothelial parameters reflect only part of the activation of endothelial cells during inflammation. We cannot exclude that we have missed effects related to other activation pathways.

In conclusion, defibrotide induced fibrinolysis during experimental endotoxemia and had modest effects on coagulation *in vivo*.

## Methods

The trial was conducted in accordance with the Good Clinical Practice guidelines and the principles set forth in the Declaration of Helsinki. The National Competent Authority (Austrian Agency for Healthy and Food Safety) and the independent ethics committee of the Medical University of Vienna approved the trial before its initiation. The project was conducted at the Department of Clinical Pharmacology at the Medical University of Vienna between April 28^th^ 2017 and February 12^th^ 2018. The trial was ended after the last subject performed the follow-up visit. The trial was registered at the EudraCT database with the public identifier 2016-001375-77 on March 24^th^ 2016 and at Clinicaltrials.gov with the public identifier NCT02876601 on June 27th 2016. Written and oral informed consent to participate in the trial was obtained from all healthy volunteers before any trial-related activity was performed. The protocol is available upon request to the corresponding author.

## Population

Eight healthy volunteers ≥18 years of age were included in a pre-study. Exclusion criteria comprised intake of medication deemed relevant by the investigators, acute illnesses and known coagulation disorders.

For the main trial twenty healthy volunteers ≥18 years of age, <90 kg body weight, with normal findings in their medical history and their physical examination, normal baseline laboratory results were included. Exclusion criteria comprised intake of any drugs that may interfere with the trial’s endpoints: acute illnesses with systemic inflammatory reactions, positive HIV or hepatitis tests, known allergies, hypersensitivities or intolerances to any of the used substances, acute or recent bleeding episodes, participation in an endotoxemia trial within six weeks of the trial start, pregnancy or breastfeeding.

## Trial Design

The pre-study was an *ex-vivo* spiking study using increasing concentrations of defibrotide performed in whole blood obtained from healthy human volunteers. Thromboelastometry was performed in these samples to detect any direct effects of defibrotide on this testing system.

**Figure 4:**
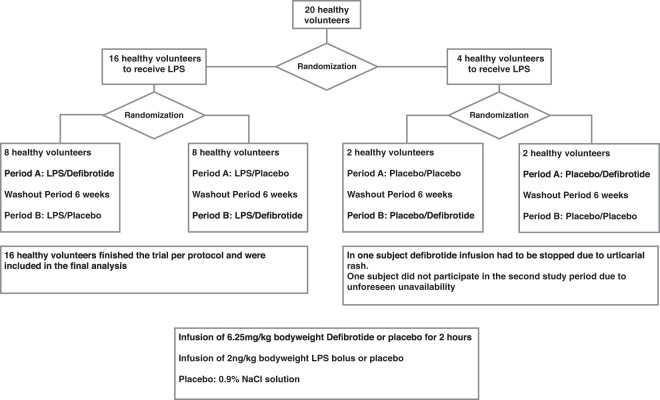
Flowchart of the trial.

The main trial was a prospective, block-randomized, double blind, placebo-controlled, two-way crossover trial in healthy volunteers. First, healthy volunteers were randomized to receive placebo or LPS. Sixteen patients were randomized to receive LPS, while four patients were randomized to receive placebo. In a second step, all subjects were randomized to receive defibrotide at a dose of 6.25 mg/kg bodyweight or placebo during the first study period. They received the respective other substance in the second study period (Fig. 4).

On the trial days subjects reported to the ward in the morning after an overnight fast. Baseline blood sampling was performed. Thereafter the infusion of 6.25 mg/kg bodyweight defibrotide or placebo was initiated for a period of 2 h. To ensure blinding defibrotide and placebo were filled in colored syringes and colored infusion lines. One hour after the start of the infusion a 2 ng/kg bodyweight LPS bolus was infused via a separate intravenous catheter. Following the infusion of LPS all subjects received a continuous infusion of 100 mL/h 0.9% saline solution until discharge. The timing of the LPS bolus was chosen to ensure relevant defibrotide levels for the critical time of the inflammatory response. To alleviate potential LPS-triggered flu-like symptoms paracetamol (500 mg tablets) was available for all subjects, which reportedly did not impact on endpoints^33^. Blood samples were drawn by fresh venipunctures at baseline (=pre), 0 h (=time of LPS bolus), 1 h, 2 h, 4 h, 6 h and 24 h thereafter. Subjects received a standardized meal four hours after infusion of LPS and were discharged from the study ward six hours after infusion of LPS. The next morning, 24 h after the LPS bolus another visit was planned. After a washout period of six weeks subjects received the alternative treatment, defibrotide or placebo. All procedures were repeated in the second study period.

### Coagulation, fibrinolysis, endothelial activation and inflammatory cytokines

Differential blood counts were performed by the ISO9002 accredited central laboratory of the General Hospital of Vienna using the Sysmex XE-2100/XE-5000^34^. To measure the effects of defibrotide on the activation of coagulation and fibrinolysis in the endotoxemia model we performed commercially available enzyme linked immunoassays (ELISA) to measure levels of prothrombin fragment F1 + 2 (F1 + 2) (Enzygnost® F1 + 2, Siemens), thrombin-antithrombin complexes (TAT) (Enzygnost® TAT, Siemens), plasmin-antiplasmin complexes (PAP) (PAP Elisa, DRG® International Inc., New Jersey, USA), tissue-type plasminogen activator (Technozym t-PA Combi Actibind Elisa kit, Technoclone, Vienna, Austria) levels and plasminogen activator inhibitor-1 (Technozym PAI-1 Actibind Elisa Kit, Technoclone, Vienna, Austria) levels. To investigate the influence of defibrotide on endothelial activation during endotoxemia we measured soluble E-Selectin by ELISA (sE-Selectin/CD62E Quantikine, R&D Systems, Minneapolis, MN, USA) and vWF antigen levels (REAADS VWF:Ag ELISA, Corgenix, Broomfied, CO, USA). Furthermore we measured pro-inflammatory cytokines including tumor necrosis factor (TNF)-α (Human TNF-α Quantikine HS, R&D Systems, Minneapolis, MN, USA) and interleukin-6 (IL-6) (human IL-6 quantikine HS, R&D Systems, Minneapolis, MN, USA). C-reactive protein levels were measured as described previously^35^. All assays were performed according to the manufacturers’ instructions.

### Thrombelastometry

Thromboelastometry (ROTEM; Pentapharm GmbH, Munich, Germany) was performed as previously described^36^. Thrombelastometry is sensitive to infusion of 2 ng/kg bodyweight LPS^36^. First, in an *ex-vivo* spiking study we investigated the concentration-dependent effects of defibrotide on the results of thromboelastometry at the doses 0 µg/mL, 25 µg/mL, 50 µg/mL and 100 µg/mL. The concentrations were chosen based on a previous publication^13^. The parameters of interest comprised the clotting time and maximum lysis.

## Endpoints

The primary endpoint of this trial was the change in plasma levels of prothrombin fragments F1 + 2. Secondary endpoints included TAT complex levels, t-PA levels, PAI-1 levels, soluble E-selectin levels, vWF antigen levels, TNF-α levels, Il-6 levels, C-reactive protein levels and differential blood counts. Furthermore we analyzed parameters of thromboelastometry: the clotting time [sec] and the maximum lysis [%] were analyzed. Safety assessments included vital parameters, documentation of adverse events and intake of concomitant medication.

### Randomization and blinding

First healthy volunteers were randomized to receive placebo or LPS. In a second step, they were randomized to receive defibrotide or placebo first. Randomization lists were generated by an independent statistician applying block randomization with block sizes of 8. Individual randomization codes and treatment allocation were concealed until immediately before administration. An unblinded study nurse under supervision of an unblinded physician who had access to treatment allocation codes prepared study drugs. They were not otherwise involved in the conduct of the trial. Participants, investigators and laboratory staff were blinded. Since defibrotide has a yellow color, both study drugs were filled in colored syringes and colored infusion lines were used to make them indistinguishable from each other. Subjects, treating physicians and other study staff who were involved in the study conduct were blinded.

### Sample size

For the *ex vivo* trial a formal sample size calculation was not performed, since no data was available for the effects of defibrotide on thromboelastometry. Therefore, a first analysis was performed after eight subjects in order to re-evaluate the sample size for the main trial based on these results.

The sample size calculation for the main trial was based on the experience with prior trials using anti-coagulants in the human endotoxemia model. Ten to 15 subjects in each group were sufficient to detect significant differences in the primary outcome parameter (F1 + 2 levels) between treatment groups in a parallel-group study^37–39^. There were no data on the expectable effect size of defibrotide in the same model. In a prior LPS trial F1 + 2 levels of approximately 819 ± 173 (Standard deviation) pmol/ml were measured^40^. This corresponds to an approximate six-fold increase in F1 + 2 levels and was similar in other endotoxemia trials^10,12,41^. The median intra-individual coefficient of variation was 15% in a recent study, the mean was 25%^41^. Thus, a sample size of 16 volunteers was deemed sufficient to detect a 25% lower increase in the defibrotide period. The placebo group consisting of four healthy volunteers formed an additional control group, which also allows to assess the effects of defibrotide in healthy volunteers without LPS. However, this analysis is exploratory and no sample size calculation for this group was performed.

### Statistical analysis

Demographics and baseline data are presented with descriptive statistics (median and quartiles).

For the analysis of the above-mentioned endpoints area under the curves (AUC) of each period normalized to the baseline (as fold increase) were calculated and compared with the non-parametric Wilcoxon test, for reasons of robustness. For the analysis of clotting time in thromboelastometry ratios compared to baseline, and in a second step deltas (difference between baseline and measuring time point) compared to baseline were calculated. Similar to other parameters, AUCs were calculated and compared. C-reactive protein levels were only measured at baseline and after 24 hours, therefore the 24 hours value was compared by the Wilcoxon test. The ratio of the AUC was calculated for both trial periods to determine effect sizes (AUC defibrotide divided by AUC placebo, medians and quartiles based on the individual ratios). Correlations were calculated by the Spearman Ranks correlation test. Adverse events and concomitant medication were documented and are reported by descriptive statistics. As this trial was of an exploratory nature, no corrections for multiple testing applied. As no corrections for multiple testing were done, even though exploratory, inferences of significance should not be made, rather p-values should be noted as nominal. Missing data were not imputed, outliers were removed from analysis.

## Supplementary information

Platelet and Leukocyte counts
