# Supplementary material for: Defibrotide enhances fibrinolysis in human endotoxemia – a randomized, double blind, crossover trial in healthy volunteers
Source: Sci Rep. 2019 Jul 31;9:11136. doi: 10.1038/s41598-019-47630-6 (PMC6668569; doi:10.1038/s41598-019-47630-6)
Supplement: Supplementary file 1 — Platelet and Leukocyte counts [file 41598_2019_47630_MOESM1_ESM.docx]

**Defibrotide enhances fibrinolysis in human endotoxemia – a randomized, double blind, crossover trial in healthy volunteers**

Christian Schoergenhofer^1^, Nina Buchtele^1^, Georg Gelbenegger^1^, Ulla Derhaschnig^1^, Christa Firbas^1^, Katarina D Kovacevic^1^, Michael Schwameis^2^, Philipp Wohlfarth^3^, Werner Rabitsch^3^, Bernd Jilma^1^

^1^ Department of Clinical Pharmacology, Medical University of Vienna, Austria

^2^ Department of Emergency Medicine, Medical University of Vienna, Austria

^3^ Department of Blood and Bone Marrow Transplantation, Medical University of Vienna, Austria

Corresponding Author:

Bernd Jilma, MD

Department of Clinical Pharmacology

Medical University of Vienna

Währinger Gürtel 18-20

1090 Vienna, Austria

Bernd.jilma@meduniwien.ac.at

Tel: +43 1 40400 29810

Fax: +43 1 40400 29980

**Running Title**: Defibrotide during endotoxemia

**Supplement:**

**Figure S1 Platelet and leucocyte counts**

**Figure S1 Platelet and leucocyte counts**

Upper Panel: fold-change in platelet counts during experimental endotoxemia (n=16 for LPS, n=4 for placebo); lower panel: fold-change in leucocyte counts during experimental endotoxemia and after placebo infusion (n=16 for LPS, n=4 for placebo); presented are medians ± interquartile range.
